# Effects of exergaming on cardiovascular risk factors and adipokine levels in women

**DOI:** 10.1007/s12576-017-0581-5

**Published:** 2017-11-30

**Authors:** Maria Guadalupe Soares Amorim, Maurício Dias de Oliveira, Daiane Santos Soares, Leandro da Silva Borges, Alexandre Dermargos, Elaine Hatanaka

**Affiliations:** 1 Instituto de Ciências da Atividade Física e Esportes, Universidade Cruzeiro do Sul, Rua Galvão Bueno, 868, 13° Andar, Bloco B, Liberdade, São Paulo, SP 01506-000 Brazil; 2 Universidade Paulista (UNIP), São Paulo, SP Brazil

**Keywords:** Exercise, Inflammation, Adiponectin, Cytokines, Xbox 360 Kinect games

## Abstract

The aim of this study was to examine the effects of* exergaming* on the cardiovascular risk factors and adipokine levels of women engaged in the music video game Just Dance using the Xbox 360 Kinect console. Triglycerides, total cholesterol, HDL, LDL, FFA, CRP, TNF-α, Il-1β, IL-6, leptin, and resistin were measured in sedentary women before and 1 month after the practice of exergaming. We also evaluated anthropometric parameters. Our results indicate that total serum cholesterol, triglycerides, and LDL were reduced by 64, 29, and 12%, respectively. HDL levels were not altered in the conditions of this study. A significant decrease was also found in the concentrations of TNF-α (57%), CRP (84.7%), resistin (68.4%), leptin (35%), FFA (90%), body mass index (10.5%), fat percentage (10.9%), weight (5.6%), abdominal circumference (2.3%), hip circumference (2.5%), and waist circumference (1.9%) after the training period. We concluded that exergaming was successful in reducing body fat, serum adipokine levels, and lipid profiles, thus reducing cardiovascular risks for women.

## Introduction

Adipocytes store triacylglycerol (fatty acids and glycerol) and play a role in integrating systemic metabolism, immune function, and dyslipidemia. Adipose tissue secretes proinflammatory adipokines [leptin, resistin, tumor necrosis factor alpha (TNF-α), interleukin 1 beta (Il-1β), and interleukin 6 (IL-6)], which contribute to a chronic low-grade inflammatory state and to metabolic disorders associated with cardiovascular disease, insulin resistance, and metabolic syndrome [1]. In obesity, TNF-α, Il-1β, and IL-6 may induce the hepatic release of C-reactive protein (CRP). CRP selectively binds apolipoprotein B (apoB)-containing lipoproteins (low-density lipoprotein, LDL, and very low density lipoprotein, VLDL). These interactions change lipid metabolism, thus altering triacylglycerol, cholesterol, LDL, and free fatty acid (FFA) levels [2]. CRP is therefore related to cardiovascular risks, dyslipidemias, obesity, and atherosclerosis, in which lipoproteins are also important [3, 4]. It is reasonable to state that exercise is one of the most common strategies used to improve health, improving the lipid and inflammatory profiles and reducing cardiovascular risk.

Exergaming, which includes Xbox 360 Kinect games, is a promising technology that uses interactive games to increase exercise behavior. In exergaming, players interact physically with onscreen avatars through body movements such as dancing, kicking, and jumping, enabling them to increase their levels of physical activity and health [5, 6]. During exergaming, this interaction of individuals with a virtual environment is triggered by their own movements, which are captured by various devices (accelerometer, force plate, cameras) and sent to the console (*Nintendo Wii, X*-*Box, PlayStation*). Exercising improves cognitive (attention, memory) [7, 8] and motor skills (postural balance, gait and executive functions) [9–12]. In recent years, exergames have been used as an exercise modality, but evidence of its effectiveness on health-related outcomes in individuals remains unclear. Dancing, and therefore, dance games such as Just Dance, elicits positive effects on physical, psychological, and social outcomes. In addition, interactive dance games have positive effects on players’ social lives and physical health, such as increasing aerobic capacity, endurance, and muscle strength [13].

The aim of this study was to evaluate the effect produced on women by the dance game Just Dance of the Xbox 360 Kinect console, based on their measured CRP, lipid profile (triglycerides, total cholesterol, and fractions), adipokines (TNF-α, IL-1β, and IL-6 leptin and resistin), FFA and anthropometric parameters (weight, body mass index (BMI), abdominal circumference, hip circumference, waist circumference, and percentage of body fat). These markers were assessed before and 1 month after beginning the practice of exergaming (1 h gaming twice a week).

## Materials and methods

### Volunteers

With the approval of the Ethics Committee of Cruzeiro do Sul University (Protocol no. 120/2014), 12 women volunteered to participate in this study. All the participants signed an informed consent form agreeing to submit to the procedures involved in the study. The group had the following characteristics (mean ± SE): age 35 ± 2 years, weight 64 ± 4.3 kg, height 1.60 ± 0.01 m, BMI 24.9 ± 1.5 kg/m^2^, lean body mass 43.5 ± 2.1 kg, body fat 23.1 ± 2 kg, percentage of body fat 31.3 ± 1.8%, and had no sports experience. Exclusion criteria for participation in the study were: a history of infection, viruses, chronic lesions, diabetes, rheumatoid arthritis, hormonal dysfunction, lupus, or other inflammatory and hematologic diseases (such as hemoglobinopathies), and taking medication. The participants danced 1 h a day, twice a week, for 1 month.

### Exercise protocol and sample collection

The Xbox Kinetic game was played twice a week for 60 min. Based on the dance game Just Dance, the protocol of the study was composed of pre-defined songs, all at the same intensity (55–69% of maximum HR). In addition, the game Just Dance has its own classification of difficulty levels, and to determine the maximum HR during the game, after a 2-week training period for adaptation, the study used some songs of the level classified as “difficult,” which required greater physical effort by the participants. Exergaming training was performed at 5 pm, twice a week. Training intensity was estimated by monitoring each participant with a Polar FT7 M heart rate monitor (HR) during exercising. At the beginning of the study, we recorded the resting HR and the maximum HR during training. The resting HR of the participants was 79.8 ± 1.3 beats per minute (bpm) and the maximum HR was 168.2 ± 2.2 bpm. According to the guidelines of the American College of Sports Medicine [14], the intensity of exergaming training was classified as moderate, and this intensity was maintained throughout the study. Exergaming was carried out between 4:00 and 5:00 pm. A limitation of this study is that we did not collect samples and analyze them considering the participants’ ovarian cycle.

The samples were collected in the fasting state. Twenty milliliters of venous blood was drawn before and after the training period. The blood samples were drawn from one of three main veins in the antecubital fossa (the cephalic, basilic, and median cubital veins). In each case, the vein was chosen based on the identification of an optimal site by both visual and tactile exploration. The blood samples were drawn into two BD Vacutainer^®^ tubes, the first containing heparin, which was used for plasma collection. The blood samples were centrifuged (400 × *g*, 10 min), and the plasma were separated from the cell components. Serum was collected and stored at –80 °C prior to determining cytokines, adipokines, and CRP by ELISA. The lipoproteins were measured no later than 72 h after collection.

### Determination of plasma adipokines

Plasma TNF-α, IL-1β, IL-6, leptin, and resistin was determined by ELISA, according to the manufacturer’s instructions (DuoSet Kit: Quantikine, R&D Systems, Minneapolis, MN, USA). A standard curve was built for each set of samples, and the cytokines were tested, yielding a correlation coefficient in the range of 0.98–0.99 [15, 16]. Whenever necessary, the samples were diluted to fall within the linear range of values required for the methods used in this study.

### Determination of plasma lipoproteins

Total cholesterol, HDL, and triglycerides were measured using colorimetric kits, following the manufacturer’s instructions (Bioclin Diagnostics, Minas Gerais, Brazil). LDL was measured with a colorimetric kit (Labtest Diagnostics, Minas Gerais, Brazil.

### Determination of free fatty acids (FFA) in plasma

NEFA kits were supplied by Wako Diagnostics (Mountain View, CA, USA). FFA was measured according to the manufacturer’s instructions. Briefly, the method involves the acylation of coenzyme A (CoA) by FFA in the presence of added acyl-CoA synthetase (ACS). The resulting acyl-CoA is oxidized by adding acyl-CoA oxidase, thus generating H_2_O_2_, which, in the presence of peroxidase, enables the oxidative condensation of 3-methy-*N*-ethyl-*N*(β-hydroxyethyl)-aniline (MEFA) with 4-aminoantipyrine to form a purple-colored adduct that can be measured colorimetrically at 550 nm. A standard curve was built for each set of samples, yielding a correlation coefficient in the range of 0.98–0.99. The lower limit of detection for the NEFA analysis was 0.016 mEq/l. Whenever necessary, the samples were diluted to fall within the linear range of values required for the methods used in this study.

### Determination of body composition

Participants’ body mass index (BMI) was calculated from their body mass and height [BMI = weight (kg)/height^2^ (m^2^)] and waist circumference was measured with an unstretchable metric tape at the midpoint between the lower border of the rib cage and iliac crest. The percentage of body fat was determined using a tetrapolar bioimpedance (Biodynamics Corporation, EUA), according to the manufacturer’s instructions.

### Statistical analysis

The values are presented as the mean ± standard error of the 12 participants. The statistical analysis consisted of one-way analysis of variance (ANOVA), using the Student–Newman–Keuls multiple comparison post hoc test (INStat; Graph Pad Software, San Diego, CA, USA) and the *t* test to correlate the samples. The significance level was set at *p* < 0.05.

## Results

**Fig. 1:**
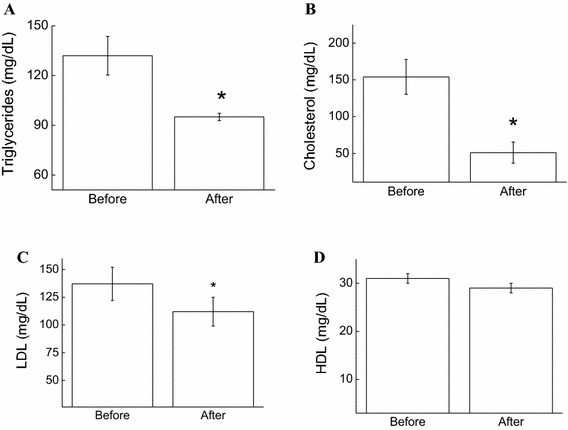
Effect of XBox 360 Kinect Just Dance exergame on lipid markers. The participants’ serum concentrations of triglycerides (**a**), cholesterol (**b**), LDL (**c**) and (**d**) HDL were determined before and 1 month after regular exergaming practice. The values are presented as mean ± standard error of the 12 participants. ^*^ *p* < 0.05 for comparison of pre- and post-training conditions

In this study, we initially evaluated the effect of exergaming on cardiovascular risk factors. Figure 1 illustrates the triglyceride (a), cholesterol (b), LDL (c), and HDL (e) levels in the blood samples of the exergamers before and after the training period. Our results demonstrate that total serum cholesterol, triglycerides, and LDL had decreased by 64 (*p* < 0.05), 29 (*p* < 0.05), and 12% (*p* < 0.05), respectively. HDL was not altered in the conditions of this study.

**Fig. 2:**
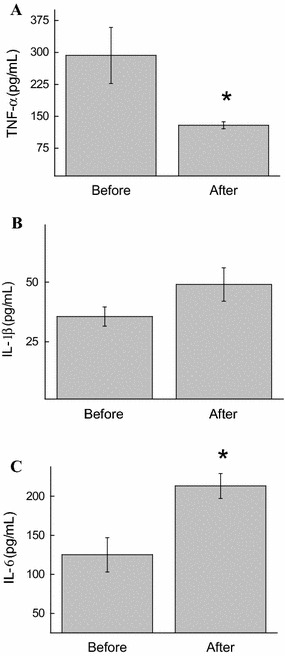
Effect of XBox 360 Kinect Just Dance exergame on the cytokine profile. The participants’ serum concentrations of TNF-α (**a**), Il-1β (**b**), and Il-6 (**c**) were determined before and 1 month after regular exergaming practice. The values are presented as mean ± standard error of the 12 participants. ^*^ *p* < 0.05 for comparison of pre- and post-training conditions

**Fig. 3:**
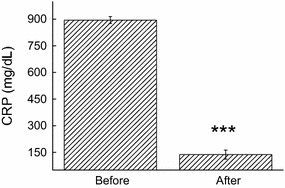
Effect of XBox 360 Kinect Just Dance exergame on CRP. The participants’ serum concentrations of CRP were determined before and 1 month after regular exergaming practice. The values are presented as mean ± standard error of the 12 participants. ^*^ *p* < 0.05 for comparison of pre- and post-training conditions

Dyslipidemia and related conditions such as obesity and insulin resistance are often accompanied by chronic low-grade inflammation. Under the study conditions, a decrease in the concentration of TNF-α (57%, *p* < 0.05) was also noted immediately after the exergame practice (Figs. 2a, 3a). Although we found no differences in the IL-1β levels (Fig. 2b), a significant decrease was observed in the concentration of CRP (84.7%, *p* = 0.01) (Fig. 3).

**Fig. 4:**
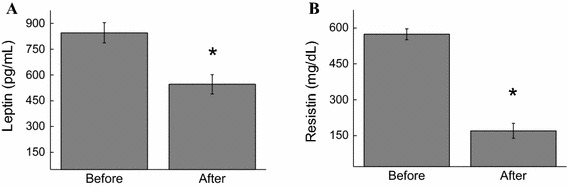
Effect of XBox 360 Kinect Just Dance exergame on adipokines. The participants’ serum concentrations of leptin (**a**) and resistin (**b**) were determined before and 1 month after regular exergaming practice. The values are presented as mean ± standard error of the 12 participants. ^*^ *p* < 0.05 for comparison of pre- and post-training conditions

Studies have been focusing in strategies to reduce low-grade inflammation [17]. Adipokines modulate the molecular events in the metabolic homeostasis of the entire body. Under the study conditions, the concentrations of resistin (68.4%, *p* < 0.01), leptin (35%, *p* < 0.05), and FFA (90%, *p* < 0.05) were also found to decrease (Fig. 4). These decreasing levels were attributed to the decrease in BMI (10.5%, *p* < 0.05) and fat percentage (10.9%, *p* < 0.05) after the exergaming period. The weight (5.6%, *p* < 0.001), abdominal circumference (2.3%, *p* < 0.01), hip circumference (2.5%, *p* < 0.001), and waist circumference (1.9%, *p* < 0.001) were also found to decrease (Table 1). The participants’ weight was 63.95 ± 4.3 (pre) and 60.37 ± 4.16 (post) and their abdominal circumference was 85.66 ± 3.66 (pre) and 83.69 ± 3.36 (post). It should be noted that although the participants were not classified as obese based on their BMI, they were considered a high-risk cardiovascular population based on their percentage of fat and abdominal circumference.

**Table 1:** Effect of XBox 360 Kinect Just Dance exergame on the anthropometric aspects

Anthropometric aspects	Pre	Post
Weight (kg)	63.95 ± 4.27	60.37 ± 4.16***
BMI (kg/m^2^)	24.85 ± 1.55	23.46 ± 1.51***
Body fat (%)	31.33 ± 1.83	27.48 ± 1.85
Abdominal circumference (cm)	85.66 ± 3.66	83,69 ± 3.36**
Hip circumference (cm)	102.95 ± 2.91	100.39 ± 2.82***
Waist circumference (cm)	76.26 ± 3.26	74.81 ± 3.27***
Waist–hip ratio (cm)	0.74 ± 0.01	0.74 ± 0.02

The participants’ weight, BMI, body fat, abdominal circumference, hip circumference, waist circumference, and waist–hip ratio were determined before and 1 month after regular exergaming practice. The values are presented as mean ± standard error of the 12 participants

** *p* < 0.01 and *** *p* < 0.001 for comparison of pre- and post-training conditions

## Discussion

Relative risk analyses have estimated the number of deaths attributable to excess weight at about 337,000 in Europe and at about 401,000 in the United States [18]. Obesity has also been shown to have a negative effect on longevity, reducing the life of obese people by an estimated 5–20 years [19]. In addition, visceral adiposity is independently associated with the incidence of cardiovascular disease [20], and even individuals with low BMI and increased abdominal obesity would be part of the higher-risk group [21]. This cardiometabolic risk related with abdominal obesity is associated with adipocytes, dyslipidemia, and systematic inflammation, which play essential roles in the pathogenesis of cardiovascular disease and metabolic syndrome [22].

Although the effects of exercise on cardiovascular risk and chronic subclinical inflammation are widely known, few studies have focused specifically on the effects of exergaming (Kinect for Xbox) on health. Exergames have shown a positive effect on health, but the literature on this subject is still scanty. Most of the papers that have been published describe the effects of exergames on postural control, attention, and motor skills. Camara Machado et al. [23], who investigated the effects of intervention based on interactive games using the motion sensor Kinect on children with cerebral palsy, concluded that Xbox 360 Kinect interactive games may be useful tools in rehabilitation interventions [23]. Several other studies have identified exergames as useful tools to improve attention, memory, postural balance, gait, and executive functions [7–13, 24].

To the best of our knowledge, this is the first study that has investigated the effects of a 1-month period of exergaming on lipid and inflammatory markers. Our results indicate that dancing regularly using digital technology improves the lipid metabolism by decreasing total serum cholesterol, triglycerides, and LDL levels. The decrease in lipid markers observed in this study can probably be attributed to the decreased concentrations of CRP, TNF-α, and IL-1β.

CRP, an acute-phase protein, is produced by the liver in response to injury and/or infection. Increased serum CRP levels have been associated with the development of atherosclerosis, ischemic attacks, hemorrhagic stroke, as well as disease outcomes. This protein is a strong predictive factor of mortality and morbidity in cardiovascular patients. CRP selectively binds apolipoprotein B (apoB)-containing LDL and VLDL and changes lipid metabolism. In vitro, CRP binds to phospholipids in liposomes [25] and modified cell membranes [26], thus altering triacylglycerol, cholesterol, LDL, and fatty acid levels [2].

In this study, we observed a significant decrease in CRP concentrations after 1 month of exergaming. Our results partially corroborate the findings of Roopchand-Martin et al. [27]. In a pilot study of sedentary female university students using the Xbox Kinect for exercise conditioning, the aforementioned authors concluded that exergaming can improve the cardiovascular conditioning and flexibility of sedentary female university students [27].

Cebolla et al. [28] studied alternative options to prescribe physical activity for obese children and adolescents, and concluded that exergaming can be a tool to help obese children in the practice of brisk walking as part of a program designed to treat obesity. The obese children scored significantly higher in expectations and satisfaction in the exergame condition, but not in self-efficacy, perceived exertion, or physiological measures [28]. Christison and Khan [29] concluded that exergaming for a health program may be an effective weight management intervention that is feasible, with high participation rates. In a recent study, Garde et al. [30] demonstrated that mobile exergames are useful tools for schools for promoting physical activity and combating obesity in adolescents and adults. In fact, active video games may offer an effective strategy to increase physical activity in overweight and obese children [6, 31–38].

Although the participants in this study were not classified as obese according to their BMI, they had a significantly higher-than-average percentage of fat and abdominal circumference [37], and are therefore considered a high-risk population for cardiovascular disease [38, 39].

Like CRP, TNF-α is considered a link between hypertriglyceridemia, inflammation, and insulin resistance [40]. Under the conditions of this study, we found that exercising using the video game approach decreased TNF-α serum levels. TNF-α levels are significantly higher in the adipose tissue of obese people. Elevated TNF-α levels induce insulin resistance by down-regulating the tyrosine kinase activity of the insulin receptor and decreasing the expression of GLUT-4 glucose transporters. TNF-α also reduces lipoprotein lipase activity in white adipocytes, and stimulates hepatic lipolysis [40].

There is a relationship between low-grade inflammatory state and leptin in obesity, suggesting that leptin could exert peripheral biological effects as a function of its cytokine-like structure [41]. Studies have also shown that there is an increased inflammatory response related with the presence of hyperleptinemia without obesity [42], and that leptin is able to control TNF-α production and activation by macrophages [42]. Another mechanism that could explain the anti-inflammatory effect of exergaming is the evidence that a marked increase in the systemic concentration of IL-6 found in response to exercise originates from the contracting limb and that skeletal muscle cells are themselves the likely source of this production. Since IL-6 inhibits low-level TNF-α production [43], it is suggested that this cytokine may have played an important anti-inflammatory role in this study.

Positive effects of regular exercise on lipoproteins generally occur with weekly energy expenditures. For instance, thresholds established from cross-sectional and longitudinal exercise training studies suggest that an energy expenditure of 1200–2200 kcal per week is associated with HDL increases of 2–8 mg/dl and triglyceride reductions of 5–38 mg/dl [44]. This study did not include a follow-up of the participants after the proposed training program and its duration was shorter than other studies that evaluated lipoproteins after traditional physical training [43–46]. However, we speculate that the increased energy expenditure generated by exergaming training [47] may result in a negative energy balance in participants and accelerate the reduction of adipose tissue and lipoprotein concentrations. Furthermore, this hypothesis is reinforced by the significant reductions in weight, BMI, and abdominal, hip, and waist circumference.

Although energy expenditure during training was not measured in this study, the level of physical activity engendered by most of the active videogames tested were moderately intense, in line with the health and fitness guidelines of the American College of Sports Medicine (from 3 to 7 metabolic equivalents, or 4–6.7 kcal expended per minute) [48, 49]. Although individual diets were not monitored, and food intervention through calorie restriction was not promoted in this study, the authors observed changes in the participants’ eating behavior. However, it is important to note that the underlying mechanisms of exergame-induced changes in cardiovascular risk factors and adipokine levels in dancers are still unknown, and moreover lie outside the scope of this study. Despite the limitations of this study, knowledge about the physical changes caused by exergaming is important in order to improve overall health.

Our findings indicate that dancing using digital technology also improves FFA, BMI, and percentage of body fat lipids. Exercise may also control leptin and resistin levels. These mediators are important for the insulin resistance of obese subjects through their over-expression in adipose tissue. Leptin secretion from circulation acts as a signal in the brain of patients with hyperinsulinemia. Additionally, white adipocytes produce resistin, a hyperglycemic hormone that blocks the insulin-stimulated uptake of glucose by adipocytes and promotes glycolysis. High levels of insulin circulating in the blood induce the secretion of resistin, leading to insulin-antagonistic effects [40, 50, 51].

The regulation of TNF-α, Il-1β, leptin, and resistin affects adipocyte insulin sensitivity and lipid accumulation, thereby improving health. Exercise training has been suggested for the prevention and treatment of such disorders [47, 48]. The results of this study demonstrate that exergaming successfully lowered the body fat and serum levels of adipokines and lipid profiles of the 12 female participants, thus indicating that it is a useful tool to reduce the risks of cardiovascular disease.

## References

[1] Mancuso P (2016). The role of adipokines in chronic inflammation. Immunotargets Ther.

[2] Rowe IF, Soutar AK, Trayner IM, Baltz ML, de Beer FC, Walker L, Bowyer D, Herbert J, Feinstein A, Pepys MB (1984). Rabbit and rat C-reactive proteins bind apolipoprotein B-containing lipoproteins. J Exp Med.

[3] Ridker PM (2016). From C-reactive protein to interleukin-6 to interleukin-1: moving upstream to identify novel targets for atheroprotection. Circ Res.

[4] Ishimaru Y, Kozuka C, Nakajima K, Sasaki T (2017). Expanding frontiers in weight-control research explored by young investigators. J Physiol Sci.

[5] Biddiss E, Irwin J (2010). Active video games to promote physical activity in children and youth: a systematic review. Arch Pediatr Adolesc Med.

[6] Zeng N, Gao Z (2016). Exergaming and obesity in youth: current perspectives. Int J Gen Med.

[7] Ben-Sadoun G, Sacco G, Manera V, BourgeoisJ König A, Foulon P, Fosty B, Bremond F, d’Arripe-Longueville F, Robert P (2016). Physical and cognitive stimulation using an exergame in subjects with normal aging, mild and moderate cognitive impairment. J Alzheimers Dis.

[8] Eggenberger P, Wolf M, Schumann M, de Bruin ED (2016). Exergame and balance training modulate prefrontal brain activity during walking and enhance executive function in older adults. Front Aging Neurosci.

[9] Endo K, Matsukawa K, Liang N, Nakatsuka C, Tsuchimochi H, Okamura H, Hamaoka T (2013). Dynamic exercise improves cognitive function in association with increased prefrontal oxygenation. J Physiol Sci.

[10] Maillot P, Perrot A, Hartley A, Do MC (2014). The braking force in walking: age-related differences and improvement in older adults with exergame training. J Aging Phys Act.

[11] Agmon M, Perry CK, Phelan E, Demiris G, Nguyen HQ (2011). A pilot study of Wii fit exergames to improve balance in older adults. J Geriatr Phys Ther.

[12] Lamoth CJ, Alingh R, Caljouw SR (2012). Exergaming for elderly: effects of different types of game feedback on performance of a balance task. Stud Health Technol Inform.

[13] Lin JH (2015). Just dance: the effects of exergame feedback and controller use on physical activity and psychological outcomes. Games Health J.

[14] American College of Sports Medicine Position Stand (1998). The recommended quantity and quality of exercise for developing and maintaining cardiorespiratory and muscular fitness, and flexibility in healthy adults. Med Sci Sports Exerc.

[15] de Moura NR, Borges LS, Santos VC, Joel GB, Bortolon JR, Hirabara SM, Cury-Boaventura MF, Pithon-Curi TC, Curi R, Hatanaka E (2013). Muscle lesions and inflammation in futsal players according to their tactical positions. J Strength Cond Res.

[16] Marin DP, dos Santos Rde C, Bolin AP, Guerra BA, Hatanaka E, Otton R (2011). Cytokines and oxidative stress status following a handball game in elite male players. Oxid Med Cell Longev.

[17] Ziemann E, Olek RA, Grzywacz T, Antosiewicz J, Kujach S, Łuszczyk M, Smaruj M, Sledziewska E, Laskowski R (2013). Whole-body cryostimulation as an effective method of reducing low-grade inflammation in obese men. J Physiol Sci.

[18] Banegas JR, López-García E, Gutiérrez-Fisac JL, Guallar-Castillón P, Rodríguez-Artalejo F (2003). A simple estimate of mortality attributable to excess weight in the European Union. Eur J Clin Nutr.

[19] Fontaine KR, Redden DT, Wang C, Westfall AO, Allison DB (2003). Years of life lost due to obesity. JAMA.

[20] Zhang C, Rexrode KM, van Dam RM, Li TY, Hu FB (2008). Abdominal obesity and the risk of all-cause, cardiovascular, and cancer mortality: sixteen years of follow-up in US women. Circulation.

[21] Dagenais GR, Yi Q, Mann JF, Bosch J, Pogue J, Yusuf S (2005). Prognostic impact of body weight and abdominal obesity in women and men with cardiovascular disease. Am Heart J.

[22] Berg AH, Scherer PE (2005). Adipose tissue, inflammation, and cardiovascular disease. Circ Res.

[23] Camara Machado FR, Antunes PP, Souza JM, Santos AC, Levandowski DC, Oliveira AA (2016). Motor improvement using motion sensing game devices for cerebral palsy rehabilitation. J Mot Behav.

[24] Eggenberger P, Wolf M, Schumann M, de Bruin ED (2016). Exergame and balance training modulate prefrontal brain activity during walking and enhance executive function in older adults. Front Aging Neurosci.

[25] Volanakis JE, Wirtz KW (1979). Interaction of C-reactive protein with artificial phosphatidyl choline bilayers. Nature.

[26] Narkates AJ, Volanakis JE (1982). C-reactive protein binding specificities: artificial and natural phospholipid bilayers. Ann N Y Acad Sci.

[27] Roopchand-Martin S, Nelson G, Gordon C, Sing SY (2015). A pilot study using the XBOX Kinect for exercise conditioning in sedentary female university students. Technol Health Care.

[28] Cebolla Martí A, Álvarez-Pitti JC, Guixeres Provinciale J, Lisón JF, Baños Rivera R (2014). Alternative options for prescribing physical activity among obese children and adolescents: brisk walking supported by an exergaming platform. Nutr Hosp.

[29] Christison A, Khan HA (2012). Exergaming for health: a community-based pediatric weight management program using active video gaming. Clin Pediatr.

[30] Garde A, Umedaly A, Abulnaga SM, Junker A, Chanoine JP, Johnson M, Ansermino JM, Dumont GA (2016). Evaluation of a novel mobile exergame in a school-based environment. Cyberpsychol Behav Soc Netw.

[31] Gao Z, Chen S (2014). Are field-based exergames useful in preventing childhood obesity? A systematic review. Obes Rev.

[32] Baranowski MT, Adamo PK, Hingle M, Maddison R, Maloney A, Simons M, Staiano A (2013). Gaming, adiposity, and obesogenic behaviors among children. Games Health J..

[33] Staiano AE, Abraham AA, Calvert SL (2013). Adolescent exergame play for weight loss and psychosocial improvement: a controlled physical activity intervention. Obesity.

[34] Kamel Boulos MN (2012). Xbox 360 Kinect exergames for health. Games Health J.

[35] Feltz DL, Irwin B, Kerr N (2012). Two-player partnered exergame for obesity prevention: using discrepancy in players’ abilities as a strategy to motivate physical activity. J Diabetes Sci Technol.

[36] Staiano AE, Abraham AA, Calvert SL (2012). Motivating effects of cooperative exergame play for overweight and obese adolescents. J Diabetes Sci Technol.

[37] Gómez-Ambrosi J, Silva C, Galofré JC, Escalada J, Santos S, Millán D, Vila N, Ibañez P, Gil MJ, Valentí V, Rotellar F, Ramírez B, Salvador J, Frühbeck G (2012). Body mass index classification misses subjects with increased cardiometabolic risk factors related to elevated adiposity. Int J Obes.

[38] Balkau B, Deanfield JE, Després JP, Bassand JP, Fox KA, Smith SC, Barter P, Tan CE, Van Gaal L, Wittchen HU, Massien C, Haffner SM (2007). International day for the evaluation of abdominal obesity (IDEA): a study of waist circumference, cardiovascular disease, and diabetes mellitus in 168,000 primary care patients in 63 countries. Circulation.

[39] Flegal KM, Graubard BI (2009). Estimates of excess deaths associated with body mass index and other anthropometric variables. Am J Clin Nutr.

[40] Halle M, Berg A, Northoff H, Keul J (1998). Importance of TNF-alpha and leptin in obesity and insulin resistance: a hypothesis on the impact of physical exercise. Exerc Immunol Rev.

[41] Aguilar-Valles A, Inoue W, Rummel C, Luheshi GN (2015). Obesity, adipokines and neuroinflammation. Neuropharmacology.

[42] Loffreda S, Yang SQ, Lin HZ, Karp CL, Brengman ML, Wang DJ, Klein AS, Bulkley GB, Bao C, Noble PW, Lane MD, Diehl AM (1998). Leptin regulates proinflammatory immune responses. FASEB J..

[43] Pedersen BK, Steensberg A, Keller P, Keller C, Fischer C, Hiscock N, van Hall G, Plomgaard P, Febbraio MA (2003). Muscle-derived interleukin-6: lipolytic, anti-inflammatory and immune regulatory effects. Pflugers Arch.

[44] Durstine JL, Grandjean PW, Cox CA, Thompson PD (2002). Lipids, lipoproteins, and exercise. J Cardiopulm Rehabil.

[45] Vasankari TJ, Kujala UM, Vasankari TM, Ahotupa M (1998). Reduced oxidized LDL levels after a 10-month exercise program. Med Sci Sports Exerc.

[46] Thompson PD, Franklin BA, Balady GJ, Blair SN, Corrado D, Estes NA, Fulton JE, Gordon NF, Haskell WL, Link MS, Maron BJ, Mittleman MA, Pelliccia A, Wenger NK, Willich SN, Costa F (2003). Exercise and physical activity in the prevention and treatment of atherosclerotic cardiovascular disease: a statement from the Council on Clinical Cardiology (Subcommittee on Exercise, Rehabilitation, and Prevention) and the Council on Nutrition, Physical Activity, and Metabolism (Subcommittee on Physical Activity). Circulation.

[47] Dutta N, Pereira MA (2015). Effects of active video games on energy expenditure in adults: a systematic literature review. J Phys Act Health.

[48] Baranowski T, Maddison R, Maloney A, Medina E, Simons M (2014). Building a better mousetrap (exergame) to increase youth physical activity. Games Health J.

[49] Sween J, Wallington SF, Sheppard V, Taylor T, Llanos AA, Adams-Campbell LL (2014). The role of exergaming in improving physical activity: a review. J Phys Act Health.

[50] Baba T, Kanda T, Yoshida A, Tsukui S, Nara M, Inukai T, Umeda T, Tamura J, Kobayashi I (2000). Reciprocal changes in leptin and tumor necrosis factor-alpha with exercise in insulin resistant rats. Res Commun Mol Pathol Pharmacol.

[51] Lombardi G, Sanchis-Gomar F, Perego S, Sansoni V, Banfi G (2016). Implications of exercise-induced adipo-myokines in bone metabolism. Endocrine.

